# Omega 3 Fatty Acid and Skin Diseases

**DOI:** 10.3389/fimmu.2020.623052

**Published:** 2021-02-05

**Authors:** Yu Sawada, Natsuko Saito-Sasaki, Motonobu Nakamura

**Affiliations:** Department of Dermatology, University of Occupational and Environmental Health, Kitakyushu, Japan

**Keywords:** ω3 polyunsaturated fatty acids, resolvin, protectin, maresin, metabolites

## Abstract

Humans are exposed to various external environmental factors. Food intake is one of the most influential factors impacting daily lifestyle. Among nutrients obtained from foods, omega-3 polyunsaturated fatty acids (PUFAs) have various beneficial effects on inflammatory diseases. Furthermore, omega-3 PUFA metabolites, including resolvins, are known to demonstrate strong anti-inflammatory effects during allergic and inflammatory diseases; however, little is known regarding the actual impact of these metabolites on skin diseases. In this review, we focused on metabolites that have strong anti-inflammatory actions in various inflammatory diseases, as well as those that present antitumor actions in malignancies, in addition to the actual effect of omega-3 PUFA metabolites on various cells.

## Introduction

The environment is fundamental for humans to live on earth and influences various physiological and pathological functions in the human body (1, 2). Food intake is an essential task for animals to drive their body and allows us to reconstruct body structure from nutrients (3). Among nutrients derived from foods, fatty acids are a component of cells and are known to control various cellular functions (4, 5). Omega-3 polyunsaturated fatty acids (PUFAs) are composed of 18 or more carbon chains, with a double bond three atoms away from the terminal methyl group, and are mainly classified into three representative lipids: α-linoleic acid (ALA), docosahexaenoic acids (DHA), and eicosapentaenoic acid (EPA). ALA is enzymatically converted to EPA and subsequently converted into DHA (6). These conversions primarily occur in the liver and are extremely limited owing to the enzyme concentration in the human body (7–9). Therefore, it is reasonable to derive DHA and EPA directly from foods and/or dietary supplements enriched in fish oils.

Omega-3 PUFAs have been known to demonstrate anti-inflammatory actions in various inflammatory diseases, including psoriasis, inflammatory bowel disease, asthma, and rheumatoid arthritis  (10–12). In recent studies, omega-3 PUFA metabolites, such as resolvins (Rvs) and maresins, have revealed potent anti-inflammatory actions. Protectins (PD) and D-series Rvs are converted from DHA by 15-lipoxygenase, whereas E-series Rvs are produced from EPA by the cytochrome P450 pathways or acetylated cyclooxygenase-2. These metabolites have strong anti-inflammatory actions in various inflammatory diseases, such as animal models of asthma (13) and colitis (14), as well as antitumor actions in malignancies; however, little is known regarding their role in skin diseases. This review focused on the therapeutic potential of omega-3 PUFA metabolites for inflammatory skin diseases, as well as antitumor actions against cutaneous tumors.

## Anti-Inflammatory Action on Immune Cells and Epithelial Cells

Reportedly, omega-3 PUFA metabolites have demonstrated various actions on immune and epithelial cells. In this section, we first reviewed the influence of omega-3 PUFA metabolites on each cell type, including dendritic cells (DCs)/macrophages, T cells/regulatory T cells (Tregs)/B cells, neutrophils, and epithelial cells, which are known to be present in the skin (**Table 1**).

**Table 1:** Cell specific action of SPMs.

	DC/Mφ	T cell/Treg/B cell	Neutrophil	Epithelial cell
RvE1	Phagocyte↑ (15–20) M2 polarization↑ (21) Migration↓ (22) (23) TNFα, IL-6, IL-8, CCL4, and CCL5↓ (20), IL-12↓ (23), Superoxide production↓ (16)	Th1/Th17 cells infiltration↓ (24) T-cell trafficking ↓ (21)	Infiltration↓ (17, 25) CXCR1/4, CCR2/6↓ (21) ROS↑ (26, 27) Phagocytosis↑ (26, 28) Apoptosis↑ (29)	Migration↑ (30, 31)
RvE3	No report	No report	Migration↓ (32, 33)	No report
RvD1	Phagocytosis ↑ (20, 34–37) COX-2 and IL-1β, IL-6↓ (38), TNFα, IL-6, IL-8, CCL4, and CCL5↓ (20) MHC II, CD40, and IL-12 ↓ (39), IL-1β, CCXL10, TNF, IL-6, CCR7↓ (40), Tnf‐α, IL‐6, IL‐1β↓ (41), IL-6 and IL-8↓ (42) Infiltration of M1 macrophages and expression of inflammatory cytokines ↓ (43) M1↓/M2 polarization↑ (44) M2 polarization↑ (45–50)	TNF-α, IFN-γ, IL-17↓ (51) Migration↓ (52) Treg↑ (34, 34, 51, 53) B cell IgM and IgG production↑ (54) B-cell IgE production↓ (55)	Apoptosis↑ (50) Infiltration↓ (36, 52, 56–61) CXCR4↓ (62) Phagocytosis↓ (63)	Epithelial barrier integrity (57)
RvD2	Phagocytosis.↑ (20, 64) TNFα, IL-6, IL-8, CCL4, and CCL5↓ (20), COX-2 and IL-1β, IL-6↓ (38) M1↓/M2 polarization↑ (44)	TNF-α, IFN-γ, IL-17↓ (51) Treg↑ (51)	Infiltration↓ (64, 65) Enhancedneutrophil access to the dermis, but prevented neutrophil-mediated damage (66)	No report
RvD3	Phagocytosis↑ (67, 68)		Migration↓ (68) Phagocytosis, ROS↑ (67)	Proliferation↑ (69)
RvD5	Phagocytosis↑ (70)			
PD1	Phagocytosis↑ (15) Inflammatory cytokine suppression (71)	Leukocyte infiltration↓ (72).	Infiltration↓ (73–75) Apoptosis↑ (74)	Anti-apoptosis (76) Anti-apoptosis (77)
MaR1	COX-2, IL-1β↓ (38), *iNos*, *IL‐1b*, *IL‐6*,*TNFa↓* (78), ROS, IL-1β, TNF-α, IL-6, and INF-γ ↓ (79), ROS↓, apoptosis↑ (80) M2 polarization, *Il-10↑* (81) Phagocytosis↑ (82)	TNF-α, IFN-γ, IL-17↓ (51) Treg↑ (51, 83)	Infiltration↓ (84, 85) Apoptosis↑ (86)	IL-6, IL-8, TNF-α, CXCL1↓ (87)

### DCs/Macrophages

DCs and macrophages play central roles in the acquired immune system to determine the direction of immune responses. As antigen-presenting cells, they take up antigens *via* phagocytic mechanisms to present antigens to T cells, to determine the direction of the immune response.

Phagocytosis is promoted by resolvin E1 (RvE1) (15–20), resolvin D1 (RvD1) (20, 34–37), resolvin D2 (RvD2) (20, 64), resolvin D3 (RvD3) (67, 68), resolvin D5 (RvD5) (70), protectin D1 (PD1) (15), and maresin 1 (MaR1) (82). For apoptotic cells, the macrophage phagocytosis is enhanced by RvE1 (15), PD1 (15), and RvD1 (34). Phagocyte-dependent bacterial clearance is enhanced by maresin 1 (MaR1) (82), RvD2 (64), RvD3 (67), and RvD5 (70).

Inflammatory cytokine production is negatively regulated by RvE1 (16, 20, 23), RvD1 (20, 38, 40–42), RvD2 (20, 38), RvD3 (71), and MaR1 (38, 78–80). RvE1, RvD1, and RvD2 suppress chemokine (C-C motif) ligand 4 (CCL4), CCL5, interleukin (IL)-6, IL-8, and tumor necrosis factor (TNF)-α induced by human monocyte-derived macrophages co-cultured with tumor cell debris (20), IL-12 by DCs stimulated with pathogen extract (23), and superoxide production by macrophages stimulated with cigarette smoke (16). In macrophages stimulated with lipopolysaccharide (LPS), RvD1, RvD2, and MaR1 suppress IL-1β and IL-6 (38, 78, 79). In contrast, MaR1 promotes the production of the inhibitory cytokine IL-10 (81).

DCs/macrophages play central roles in antigen presentation, as well as migration into antigen-presenting sites for T cells to initiate their active forms. RvD1 suppresses antigen presentation by suppressing major histocompatibility complex (MHC) class II and CD40 expression (39). Additionally, DC migration and infiltration into inflamed tissues are negatively regulated by RvE1 (22, 23) and RvD1 (43). RvE1 suppresses the migration of DCs in the skin and into draining lymph nodes, attenuating the acquired immune response (22), while RvD1 attenuates the increased infiltration of M1 macrophages (43).

Owing to the characteristics of antigen-presenting cells as a control tower in the immune response, the polarization of macrophages determines the direction of inflammatory responses. M1 macrophages are induced by interferon (IFN)-γ, a microbial component. M1 macrophages produce inflammatory cytokines and induce effector cells in polarized Th1 responses. M1 polarization is reportedly suppressed by RvD1 (44) and RvD2 (44). M2 macrophages are generated during enhanced Th2 reactions, promoting parasite killing (88), tissue repair (89), and immunoregulatory functions (90). M2 polarization is positively regulated by RvD1 (44–50), RvD2 (44), and MaR1 (91).

### T Cells/Tregs/B Cells

T cells can respond to pathogens by direct contact with the antigen derived from the pathogen and need to migrate to sites in the presence of the antigen. Reportedly, T cell migration is regulated by RvE1 (21, 24), RvD1 (52), and PD1 (72). RvE1 decreases the infiltration of Th1 and Th17 cells  (24). RvE1 suppresses T cell infiltration by decreasing the production of RANTES in vascular smooth muscle cells (21).

After the initiation of immune responses by antigen-presenting cells, naïve T cells develop T cell responses as an appropriate direction of inflammatory immune responses. RvD1, RvD2, and PD1 suppress inflammatory cytokine production (51). RvD1, RvD2, and PD1 reduce Th1 and Th17 cytokine production (51).

Tregs are promising cells for the maintenance of immune homeostasis and tolerance (92). T cell‐mediated autoimmune diseases and allergies are closely related to their deficiency or dysfunction. RvD1, RvD2, and PD1 are known to contribute to the inhibition of immune reactions by increasing Tregs (51).

B cells play an important role in the adaptive immune system. The activation of B cells is induced following antigen recognition, differentiating to form plasma cells for antibody secretion. RvD1 influences immunoglobulin production by B cells and increases IgM and IgG production (54), as well as reduces IgE production (55).

### Neutrophils

Neutrophils have various functions, including phagocytosis and antimicrobial peptide production (93). Reportedly, neutrophil phagocytosis is positively regulated by RvE1 (26, 28), RvD1 (63), and RvD3 (67). In phagocytes, NADPH oxidase is essential for neutrophil microbicidal activity (94). Furthermore, reactive oxygen species (ROS) generation in neutrophils is promoted by RvE1 (26, 27) and RvD3 (67). In addition, neutrophil migration is suppressed by RvE1 (17, 25), RvE3 (32, 33), RvD1 (36, 52, 56–61), RvD2 (64, 65), RvD3 (68), PD1 (73–75), and MaR1 (84, 85). RvE1 decreases chemokine receptor expression, including C-C chemokine receptor type 2 (CCR2), CCR6, chemokine (C-X-C motif) receptor 1 (CXCR1), and CXCR4 (21), as well as chemotaxis (26). RvD1 decreases CXCR4 expression (62). Moreover, RvD2 allows neutrophils to enhance their access to the dermis; however, RvD2 demonstrates a protective role against neutrophil-mediated damage (66).

Apoptotic neutrophils progress toward secondary necrosis mediated by the release of caspase 3-processed danger signals (93). Neutrophil apoptosis is reportedly enhanced by RvE1 (29), RvD1 (50), PD1 (74), and MaR1 (86). These metabolites enhance apoptotic cell phagocytosis of macrophages, suppressing secondary necrosis-mediated inflammation.

### Epithelial Cells

Epithelial cells act as the first line of defense against the external environment. As the outermost organ layer, epithelial cells induce inflammatory cytokine and chemokine production to amplify the response to external stimuli. Inflammatory cytokine production is negatively regulated by MaR1 (87). Furthermore, MaR1 suppresses the production of CXCL1, IL-6, IL-8, and TNF-α by bronchial epithelial cells (87).

As the outer organ layer, epithelial cells exhibit migration and proliferation to shield the defect of the first line of defense against external stimuli. RvE1 enhances epithelial cell migration (30, 31), RvD3 promotes lung epithelial cell proliferation (69), while RvD1 and PD1 exhibit protective effects toward epithelial cells. Furthermore, RvD1 promotes epithelial barrier integrity (57). PD1 is shown to afford protection against repetitive oxidative stress-induced apoptosis (76, 77).

## Different Functions of PUFA Metabolites on Immune Cells and Epithelial Cells

There are some different effects of PUFA metabolites in immune cells and epithelial cells. RvE1 suppresses immune cell migration (22, 23) while it promotes epithelial cell migration (30, 31). In addition, PD1 promotes apoptosis of neutrophils (74); however, it enhances anti-apoptosis in epithelial cells (76, 77). Although the detailed mechanism remains unclear, a possible other point of action might exist in each different type of cell, because they are located in different body sites and surface layers, which are given the appropriate role in the body.

## The Anti-Inflammatory Action of PUFA Metabolites on Skin Diseases

Several reports have highlighted the anti-inflammatory actions of omega-3 PUFA metabolites on inflammatory skin diseases, including psoriasis, atopic dermatitis, contact hypersensitivity, and ultraviolet (UV) radiation. Furthermore, the antitumor effects of PUFA metabolites on squamous cell carcinoma and melanoma have been reported (**Table 2**).

**Table 2:** Anti-inflammatory and anti-tumor action of Omega-3 PUFA metabolites in skin diseases.

Inflammatory skin disease	Omega-3 PUFA metabolites
Psoriasis	MaR1 (95) RvE1 (96) RvD1 (97)
Atopic dermatitis	RvE1 (98)
Contact dermatitis	RvE1 (22)
UV radiation	MaR1 (99)
Wound healing	PD1 (48) RvD1 (100)
Malignancy	
Squamous cell carcinoma	RvD2 (101)
Malignant melanoma	RvD1 (20) RvD2 (20)

### Psoriasis and PUFA Metabolites

Psoriasis is a representative inflammatory skin disease characterized by scaly erythematous plaques with epidermal hyperplasia (102). Although the underlying mechanism of psoriasis remains unclear, recent studies have revealed some predominant pathways underlying pathological conditions, as well as the contribution of the TNF/IL-23/IL-17 axis (103). Current biologics targeting IL-17, IL-23, and TNF-α play critical roles in the pathogenesis of psoriasis (104).

Reportedly, MaR1 impairs imiquimod-induced psoriasis-like skin inflammation and IL-23 subcutaneous injection-induced skin inflammation (95). MaR1 decreases lymphocyte and neutrophil infiltration, dermal edema, and epithelial hyperplasia. MaR1 inhibits the production of IL-17A by CD4^+^ and γδTCR^mid+^ cells. Consequently, MaR1 attenuates IL-23 receptor expression on CD4^+^ and γδTCR^mid+^ cells by inhibiting retinoic acid-related orphan receptor gamma t (RORγt) in clathrin-dependent IL-23 receptor internalization.

RvE1 impairs imiquimod-induced psoriatic dermatitis (96). Furthermore, IL-17-producing cells and neutrophils are reduced in the skin following RvE1 treatment. Reportedly, IL-23 and IL-17 are downregulated by RvE1. IL-23 production by DCs, as well as the migration of DCs and IL-17 producing cells, is suppressed by RvE1.

RvD1 reduces acanthosis and hyperkeratosis induced by imiquimod (97). Inflammatory cell infiltration into the dermis is reduced following treatment with RvD1. Consistently, IL-17, IL-23, and TNF-α are decreased by RvD1.

### Atopic Dermatitis (AD) and PUFA Metabolites

AD is a representative Th2-mediated chronic inflammatory skin disease characterized by inflamed and irritative itchy skin inflammation (105). Several factors are involved in the various environmental factors that drive Th2 dominant skin inflammation, including skin barrier disruption (106, 107), pathogens (108), and prostanoids (109). In NC/Nga mice, RvE1 demonstrates anti-inflammatory actions in repeated hapten application-induced AD-like skin inflammation (98). RvE1 suppresses IL-4 and IFN-γ production by T cells, as well as serum IgE levels, and reduces the infiltration of eosinophils, mast cells, and T cells in skin lesions.

### Contact Dermatitis and PUFA Metabolites

Contact dermatitis is a common cutaneous allergic reaction that depends on the acquired immune response (110, 111). RvE1 impairs the inflammatory response in contact hypersensitivity during the sensitization and elicitation phases (22). During the sensitization phase, RvE1-treated mice exhibit significantly reduced DC migration into draining lymph nodes, subsequently reducing the number of central and effector memory T cells. Consistently, antigen-specific T cell proliferation and IFN-γ production are reduced by RvE1. In the elicitation phase, RvE1 impairs DC cluster formation, which is essential for the development of elicitation phase inflammation, subsequently suppressing the number of IFN-γ–producing CD8^+^ T cells in the skin.

### UV Radiation and MaR1

UV radiation is a representative environment-related skin inflammation that causes acute inflammation characterized by skin inflammation after sun exposure (112). MaR1 suppresses skin swelling as well as macrophage infiltration induced by UVB irradiation (99). Furthermore, MaR1 inhibits UVB irradiation-induced keratinocyte apoptosis, production of inflammatory cytokines, IL-1β and TNFα, and oxidative stress.

### Wound Healing and PUFA Metabolites

Damage induced by external factors destroys the skin surface, resulting in skin defects that appear as a wound. As the skin acts as a barrier against the external environment, wounds allow outside pathogens and irritants to infiltrate the body and cause skin inflammation (113). PD1 and RvD1 promote skin wound healing (48, 100). In diabetic wounds, RvD1 enhances macrophage phagocytosis, promoting wound closure owing to the reduced number of apoptotic cells (48). Furthermore, PD1 promotes wound closure (100). Skin wounds promote the synthesis of PD1 in the skin; however, in diabetes, the skin suppresses PD1 production. Macrophages are one of the main sources of PD1 in skin wounds and are known to contribute to the development of inflammation and oxidative stress reactions during acute inflammation in diabetic wounds.

### Antitumor Effects of PUFA Metabolites

Squamous cell carcinoma is a keratinocyte-derived malignancy, and the advanced clinical stage of this malignancy remains refractory to current systemic treatments (114). RvD2 suppresses squamous cell carcinoma development (101) and decreases inflammatory chemokines and cytokines, including CXCL10, IL-6, monocyte chemoattractant protein-1 (MCP-1), and TNF-α, by cancer cells. In squamous cell carcinoma, RvD2 decreases the infiltration of neutrophils and suppresses myeloperoxidase (MPO) activity. Additionally, RvD2 enhances the M2 macrophage population and their efferocytosis.

Malignant melanoma is a malignancy derived from melanocytes with severe life-threatening clinical behavior owing to a lack of radical treatment (115). RvD1 and RvD2 suppress melanoma development (20). Furthermore, RvD1 or RvD2 inhibit lung metastasis of melanoma cells and regulate the production of chemokines and cytokines, including CCL4, CCL5, IL-6, IL-8, and TNF-α, by human macrophages, providing antitumor immunity.

## Clinical Trial of Omega3 PUFA for Cutaneous Skin Diseases

Finally, we reviewed the clinical trials of omega 3 PUFAs for cutaneous skin diseases. There are several trials for atopic dermatitis and psoriasis.

The intake of omega-3 supplement improves the Scoring in Atopic Dermatitis (SCORAD) score (116). A double-blind, randomized, placebo-controlled trial showed AD patients who received daily ω3 fatty acid supplementation (fish oil, 10%; 200 mL) show high serum EPA concentration and a decreased disease severity of AD (117).

Psoriasis patients with obesity received energy-restricted foods enriched of ω3 PUFAs (average 2.6 g/d), and these patients showed impaired Psoriasis Area Score Index (PASI) score and Dermatological Life Quality Index (118). A double-blind, randomized study in multicenter trials showed the group of intaking daily an ω3 fatty acid (Omegavenous; 200 ml/day with 4.2 gm of both EPA and DHA) decreased total PASI score without serious side effects (119).

Although there have been no clinical trials of cutaneous malignancies, head and neck squamous cell carcinoma has been reported. Supplementation of daily 2 g EPA intakes impairs the production of serum pro-inflammatory cytokines, reduction of body weight and lean body mass, and increases quality of life in patients with squamous cell carcinoma in head and neck (120).

## Conclusion

There are a limited number of research and clinical trials for investigations of the effects of ω3 PUFA in skin diseases. According to previous studies assessing immune cells, various beneficial effects are expected in skin diseases. Furthermore, there are few reports available on keratinocytes, which are a major component of cells present in the epidermis. The detailed action of keratinocytes in the skin needs to be clarified in future research. Because the incidences of inflammatory skin diseases and malignancies are currently increasing, future basic research and clinical trials for ω3 PUFAs in dermatology fields are expected give us a beneficial information for the daily clinical treatment for skin diseases.

## Author Contributions

YS and MN wrote this manuscript, and NS-S conducted a critical review of this paper. All authors contributed to the article and approved the submitted version.

## Conflict of Interest

The authors declare that the research was conducted in the absence of any commercial or financial relationships that could be construed as a potential conflict of interest.
